# Monitoring of Auditory Function in Newborns of Women Infected by SARS-CoV-2 during Pregnancy

**DOI:** 10.3390/children10020194

**Published:** 2023-01-20

**Authors:** Enrico Apa, Maria Teresa Presutti, Cecilia Rossi, Maria Federica Roversi, Salvatore Neri, Giancarlo Gargano, Giovanni Bianchin, Valeria Polizzi, Valeria Caragli, Daniele Monzani, Alberto Berardi, Silvia Palma, Elisabetta Genovese

**Affiliations:** 1 Otorhinolaryngology Unit, Department of Medical and Surgical Sciences for Children and Adults, Azienda Ospedaliero-Universitaria of Modena, 41125 Modena, Italy; 2 Infectious Disease Unit, Department of Medical and Surgical Sciences, Sant’Orsola-Malpighi Hospital, University of Bologna, 40100 Bologna, Italy; 3 Neonatal Intensive Care Unit, Department of Neonatal Intensive Care Unit, Azienda Ospedaliero-Universitaria of Modena, 41125 Modena, Italy; 4 Department of Obstetrics and Paediatrics, Santa Maria Nuova Hospital, Centre for Clinical and Basic Research (IRCCS), 42121 Reggio Emilia, Italy; 5 Department of Audiology, Santa Maria Nuova Hospital, Centre for Clinical and Basic Research (IRCCS), 42123 Reggio Emilia, Italy; 6 ENT, Department of Surgical Sciences, Dentistry, Gynaecology and Paediatrics, University of Verona, Borgo Roma Hospital, 37100 Verona, Italy; 7 Audiology, Primary Care Department; AUSL of Modena, 41100 Modena, Italy

**Keywords:** hearing loss, SARS-CoV-2, Auditory Brainstem Evoked Response, children, healthcare policy

## Abstract

Background: Gestational SARS-CoV-2 infection can impact maternal and neonatal health. The virus has also been reported to cause newborn sensorineural hearing loss, but its consequences for the auditory system are not fully understood. Objective: The aim of this study was to evaluate the impact of maternal SARS-CoV-2 infection during pregnancy on newborn’ hearing function during the first year of life. Methods: An observational study was conducted from 1 November 2020 to 30 November 2021 at University Modena Hospital. All newborns whose mother had been infected by SARS-CoV-2 during pregnancy were enrolled and underwent audiological evaluation at birth and at 1 year of age. Results: A total of 119 neonates were born from mothers infected by SARS-CoV-2 during pregnancy. At birth, five newborns (4.2%) presented an increased threshold of ABR (Auditory Brainstem Evoked Response), but the results were confirmed only in 1.6% of cases, when repeated 1 month later, while the ABR thresholds in all other children returned to normal limits. At the 1-year follow-up, no cases of moderate or severe hearing loss were observed, while concomitant disorders of the middle ear were frequently observed. Conclusions: Maternal SARS-CoV-2 infection, regardless of the trimester in which it was contracted, appears not to induce moderate or severe hearing loss in infants. It is important to clarify the possible effect of the virus on late-onset hearing loss and future research is needed.

## 1. Introduction

Since the Severe acute respiratory syndrome coronavirus 2 (SARS-CoV-2) outbreak spread worldwide, the international scientific community has been particularly concerned about vulnerable populations, such as pregnant women and newborns. SARS-CoV-2 has been shown to be more than a respiratory viral infection, with effects being observed in many other organs and systems [1]. Vertical transmission of this virus infection has been a controversial topic and its potential pathogenetic mechanisms are not fully understood. The expression found in maternal–fetal interface tissues of angiotensin-converting-enzyme 2 (ACE-2), one of the principal SARS-CoV-2 receptors, led some authors to hypothesize that the virus may spread through the placenta, thus suggesting potential intrauterine transmission [2,3,4]. At present, the potential consequences of the infection on the fetus remain unclear [5,6]. Considering the neurotrophic properties of SARS-CoV-2 [7,8,9] and its assumed role in cochlear symptoms in adult populations [10,11,12], its spread through the inner ear and subsequent cellular damage seem conceivable [13]. Moreover, a study on animals has shown the susceptibility of the middle ear to this Coronavirus [14].

As a consequence, the possibility of onset of hearing impairment in newborns, after the mother’s viral exposure during pregnancy has been considered. This topic has been targeted by a few studies. In one of the first papers, neonates born to COVID-19-positive mothers did not seem to have an increased risk of hearing loss [15]; a few authors obtained the same results [16,17], while Alan et al. observed that SARS-CoV-2 PCR positivity in pregnancy was associated with abnormal newborn hearing screening (NHS) results [18]. On the other hand, Oskovi-Kaplan et al. observed no significant difference in hearing screening test between newborns of mothers infected by SARS-CoV-2 during pregnancy and a healthy control group [19].

Most of these authors concluded that more large-scale, multicenter studies of pregnant women were needed and more information concerning long-term follow-up of auditory function in newborns was necessary. Another aspect that is not clear is whether the consequences may be different depending on the trimester of pregnancy in which maternal SARS-CoV-2 infection appears [19,20].

In our region, a law [21] has introduced a two-stage hearing screening protocol: otoacoustic emissions (OAE) test on the second day after birth or before discharge for well babies and both the OAE test and the ABR (Auditory Brainstem Responses) for children with audiological risk factors, according to the recommendations of the Joint Commette on Infant Hearing (JCIH) [22].

An audiological surveillance program, as recommended in [22], has been planned jointly with family pediatricians. The monitoring of language development is periodically provided by pediatricians together with a suggested audiological evaluation once a year at least until 3 years of age. The adherence to the audiological program is recommended to each family with children at risk of hearing loss [21].

The aim of this study was to evaluate the impact of maternal SARS-CoV-2 infection during pregnancy on a newborn’s hearing function during the first year of life.

## 2. Materials and Methods

### 2.1. Participants

An observational study was conducted from 1 November 2020 to 30 November 2021 at University Modena Hospital when, according to general protocols, all women that accessed the hospital to give birth underwent a nasopharyngeal swab to exclude SARS-CoV-2 infection.

All newborns whose mothers had been infected by SARS-CoV-2 during pregnancy were enrolled. Women were considered infected in the case of SARS-CoV-2 positivity confirmed by reverse transcription–polymerase chain reaction (RT-PCR) on nasopharyngeal swabs. Newborns whose mothers had been infected by SARS-CoV-2 up to 14 days before the delivery were also tested with an RT-PCR test on nasopharyngeal swabs. Neonatal positivity and related symptoms were eventually acquired.

Newborns with risk factors for hearing loss such as syndromic features (e.g., atresia auris and facial dysmorphia), TORCH infections, meningitis, encephalitis, familiar history of hearing loss, aminoglycoside or other ototoxic drugs administration for more than 5 days, hyperbilirubinemia treated with exchange transfusion, neonatal intensive care unit (NICU) admission for more than 5 days, birth weight <1500 g and gestational age <28 weeks, were excluded [22].

Clinical data concerning the general course of pregnancy, age at delivery, trimester of SARS-CoV-2 infection and relative phenotype (asymptomatic vs. symptomatic), weeks of gestational age at birth, birth weight, gender, Apgar score at 1′ and 5′ min, presence of peripartum complications and acute respiratory distress were collected. 

### 2.2. Procedures

The study protocol was scheduled within two time-points: the first step at birth in the Neonatal Department and the second step within 3 months-old in the Audiology Department. All neonates underwent NHS by means of otoacoustic emissions (OAE) at birth [21]. For this test, a Madsen AccuScreen device (Natus^®^ Medical Incorporated, Taastrup, Denmark) was used. As usual, results were binary for each ear, “pass” in the case of the presence of an OAE response in both ears, and “refer” in the case of a repeated unclear unilateral or bilateral response.

Then, within the third month of life, every newborn was evaluated in the Audiology Department, where otoscopy, ABR and impedance tests were performed bilaterally. ABR was recorded during spontaneous sleep, using Medelec^®^ Synergy software. Acoustic wide range click stimuli of 21 pps were applied by headphones. Action potentials were detected using vertex-mastoid ipsilateral derivations. At least two runs were obtained at any stimulus intensity and compared to each other, in order to assess waveform repeatability. The exam was conducted presenting medium intensity stimuli, then decreasing gradually until the V wave peak threshold was obtained; lastly, a high intensity stimulus was presented to detect wave’ latencies. With regards to ABR results, the following parameters were evaluated: identification of I, III and V wave peaks at different stimulus intensity and their replicability; identification of V wave threshold expressed in dB nHL; measurements of peak latencies of I, III and V waves expressed in ms; interaural difference of V wave latency (IT-5) expressed in ms.

Thresholds of V wave identification ≤30 dB nHL, without pathological delay of latency, were considered indicative of normal results.

An acoustic immittance test was conducted in order to rule-out potential overestimations of the auditory threshold caused by middle or external ear dysfunctions. A Madsen Zodiac device (Natus^®^ Medical Incorporated, Denmark) at 1000 Hz probe tone frequency was used to test acoustic immittance. Tympanograms were classified according to the Jerger classification and acoustic reflex (AR) measures were taken using the same instrument [23].

All families with normal results were invited, as usual practice for children at risk for hearing loss, to take their children to the hospital for repeat audiological evaluation at the age of 1 year. Basically, all children underwent visual reinforcement audiometry (VRA) using a two-channel diagnostic audiometer (Piano Plus VRA, Audiology and Balance, Inventis Srl, Padova, Italy) and an immittance test.

The severity of sensorineural hearing loss was defined according to the WHO classification: mild (≥26 to <40 dB), moderate (≥41 to <55 dB), moderate-severe (≥56 to <70 dB), severe (≥71 to <90 dB) and profound (>90 dB) [24].

### 2.3. Statistical Analysis

All data were collected in a Microsoft Excel^®^ database. A descriptive analysis of variables was performed; quantitative and qualitative variables were expressed as means with standard deviations (SD) and rates. IBM SPSS Statistics^®^ software was used for analysis and graphical presentations. A chi-square test for qualitative variables and a one-way ANOVA for quantitative variables were performed. The comparison between the mean V wave threshold of the sample and the value considered normal was performed descriptively, taking into account the number of ears in which a value considered pathological was observed.

The casuistry was divided into three groups according to the trimester of maternal SARS-CoV-2. Due to the small size of each group, a bootstrapping resampling procedure for 10,000 sub-samples with Bonferroni’s post hoc analysis was applied. A *p*-value < 0.01 was considered statistically significant.

## 3. Results

### 3.1. Participants 

In the period from 1 November 2020 to 30 November 2021, a total of 3150 neonates were born; 134 (4.25%) were born from mothers infected by SARS-CoV-2 during pregnancy, among which, 15 cases were excluded due to the presence of other risk factors for hearing loss. Thus, a final sample of 119 (3.78%) newborns was identified. Epidemiological data regarding mothers and newborns are reported in Table 1. In most cases, maternal infection occurred in the third trimester. Ten newborns (9%) were admitted to the NICU for non-invasive ventilation of no more than 5 days. During the SARS-CoV-2 infection, 87 (73.1%) women reported at least one symptom (fever, cough and anosmia were the most common), while 32 (26.9%) were asymptomatic. Only in two newborns (1.7%) RT-PCR test on nasopharyngeal swabs resulted positive for SARS-CoV-2 and one (0.8%) presented symptoms. Thirteen pregnancies were not physiological (gestational diabetes, intrauterine growth restriction, pre-eclampsia and single fetal demise in twin pregnancy), while, in 14 deliveries, a peri-partum complication occurred (abnormalities in the cardiotocographic trace, premature rupture of membranes, post-partum hemorrhage or anomalies of the placenta).

### 3.2. Newborn Hearing Function

**Figure 1:**
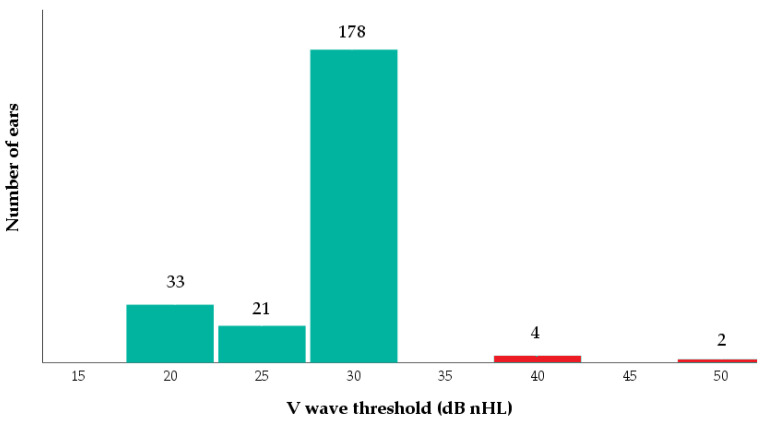
V wave threshold in all ABR measurements performed within the third month of age.

All newborns obtained a “bilateral pass” result for OAEs at newborn hearing screening. The mean gestation age at the date of ABR was 50.06 (±7.75) weeks. From the sample of 119 newborns, a total of 238 ears were considered. In 232 cases (97.5%), the V wave threshold was ≤30 dB nHL, whereas in six cases (2.5%), it was considered pathological (Figure 1). The mean value was 28.51 (±4.35) dB nHL, while the mean IT5 was 0.22 (±0.20) ms. For both SARS-CoV-2 positive infants, a normal V wave threshold was observed.

A total of five newborns (4.2%) presented a threshold elevated in ABR (Table 2), globally six tests. Only in two cases (case 3 and case 4; 1.6%), the results were confirmed when repeated 1 month later, while the threshold in all other children was normalized.

**Figure 2:**
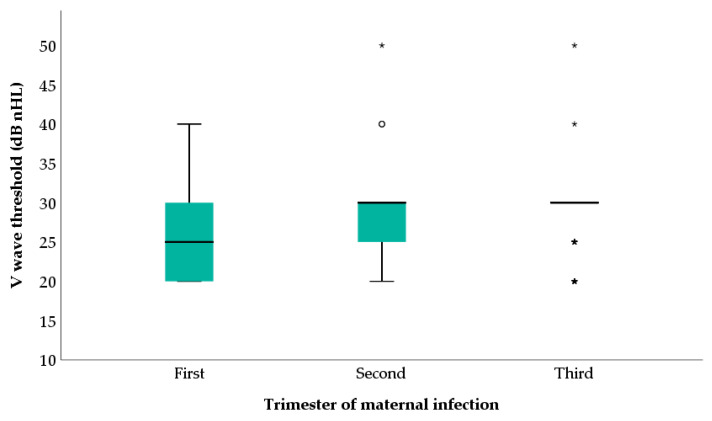
V wave threshold according to the trimester of maternal infection. Each box is included between the first and third quartiles; the box’s height is equivalent to the inter-quartile range (IQR) and contains 50% of the measurements. Values that deviate from the box by more than 1.5 IQR upward or downward are considered potential outliers and represented with * or °.

The mean V wave threshold was 25.71 (±6.46) dB nHL in neonates born from mother infected by SARS-CoV-2 in the first trimester, whereas it was slightly higher in newborns whose mother was infected in the other two trimesters. Specifically, it was 28.50 (±4.58) dB nHL and 28.81 (±3.84) dB nHL for the second and the third trimester respectively (Figure 2).

According to the one-way ANOVA test, the differences among these subgroups were not significant (*p* = 0.040). Bonferroni’s post-hoc analysis confirmed a non significant difference in fact, *p =* 0.077 and 0.034 for first-trimester infection versus second-and third-trimester maternal infection, respectively. No difference was observed between the second and the third trimester maternal infection’ subgroups (*p* = 1.000).

**Figure 3:**
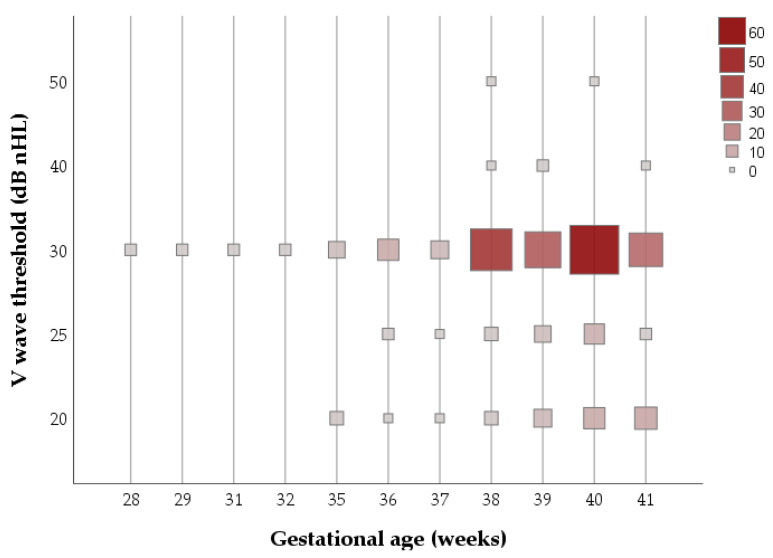
V wave thresholds of the sample, according to gestational age in weeks.

The V wave threshold was not significantly different regarding the gestational age (f = 0.927; *p* = 0.509) and the weight at birth (f = 1.831; *p* = 0.345), as shown in Figure 3.

Considering the total number of neonates from November 2020 to November 2021, the prevalence of a pathological V wave threshold in newborns from mothers infected by SARS-CoV-2 during pregnancy, at first ABR, was 1.6:1000 neonates, while the results of the subsequent ABR indicated a prevalence of 0.6:1000 neonates.

### 3.3. Follow-Up at 1 Year

Of the total sample, 37 babies (30.3%) underwent a follow-up examination at 1 year of age (Table 3). The audiological evaluation identified hearing function within normal limits. In detail, the mean PTA was 26.61 dB (*t* = −7.87; *p* < 0.000). A frequent occurrence of middle ear disorders was observed (Table 2). 

The results of the 1-year follow-up, considering only 30.3% of the sample, reported the frequent presence of concurrent disorders of the outer and middle ear. According to free-field VRA a threshold higher than 32.5 dB was never observed. Only one child with an altered threshold completed the follow-up at 1 year of life, with a good performance.

## 4. Discussion

The SARS-CoV-2 pandemic has caused millions of cases worldwide, but it has been evidenced that children infected remain mostly asymptomatic or with mild symptoms. [25,26]. Various explanations have been given, such as the simultaneous presence of other viruses in the respiratory mucosa that may compete with SARS-CoV-2 [27] or a lower ACE2 expression in the nasal epithelium in children [28]. How this virus interacts with newborns is still an object of study, even though it has been demonstrated that there is no apparent difference in growth and neurodevelopment in the first 6 months of age among infected and non-infected babies born to SARS-CoV-2 positive mothers [29].

Whether positive pregnant women will affect the hearing of their newborns remains unclear, but it is supposed that the auditory system of the newborn could be affected through intrauterine hypoxia and vertical transmission [30].

Although it seems reasonable to hypothesize that the fetal inner ear cells are damaged after the mother’s viral exposure during pregnancy, with subsequent hearing impairment, the results of our study did not strongly confirm this assumption. In fact, even if around 4% of our cases demonstrated a moderate change in the threshold of ABR, in most cases, it was retrieved, and early clinical pictures characterized by moderate or severe hearing loss in those newborns, were not evidenced.

In a recent review it was reported that SARS-CoV-2 might have a greater influence on hearing loss in newborns during the second and third trimesters of pregnancy [30]. In our statistical analysis, according to the one-way ANOVA, no significant difference was observed in the V wave threshold according to the trimester of maternal SARS-CoV-2 infection; hence, the V wave threshold was only slightly higher in newborns whose mothers had been infected in the last two trimesters, but a significant difference was not observed.

On the other hand, according to serology studies, the reported number of laboratory-confirmed cases of SARS-CoV-2 infection in children was likely underestimated given the high proportion of mild and asymptomatic cases in which testing may not have been performed [31,32]. Moreover, SARS-CoV-2 variants appear to have a different tropism [33].

Another aspect that makes it difficult to clarify the effects of the virus on newborn’ hearing is that early audiological diagnosis is not always reliable and not all children identified as suffering from sensorineural hearing loss will have permanently impaired hearing thresholds [34]. On one hand, the maturation of the auditory pathways is affected by individual variations; on the other hand, the neurotrophic properties of SARS-CoV-2 [35] could further interfere with the development of auditory processing.

At the moment, it is not possible to have studies showing data concerning prolonged follow-up since the pandemic started in 2020. In this way, the monitoring period for newborns with hearing loss should be extended to detect possible late-onset hearing impairments caused by SARS-CoV-2 infection during pregnancy [15]. It must be remembered that VRA does not present precise information about the threshold of each ear; thus, it would be necessary for children to undergo ABR which is complicated at this age and a cause of parental anxiety. Moreover, such a burden would not be easily sustainable for the health system.

In our sample, all children that came for follow-up at 1 year of age demonstrated mild/moderate conductive hearing loss. Unfortunately, only 30 % of the children completed 1 year of follow-up. This could be due to the effect of the pandemic on the national health system as all services, including essential ones, have been impacted [36], as well as to the lack of perception of the risk, especially if the development of precursors of language appears normal. Moreover, our regional health system leans on the territorial network of pediatricians which can assure a narrow follow up of children development. Lastly, some families missed the appointment because the child presented with other upper respiratory tract infections or acquired SARS-CoV-2 infection during the follow-up period. It would have been interesting to compare their hearing outcomes with controls to assess a higher percentage of conductive hearing loss; this will be the object of a future study.

This study had some other limitations. Data concerning the mothers’ vaccination status were lacking, and the exact serotype of virus responsible for maternal infection was not known. A relatively small number of children completed the audiological surveillance pathway.

The strengths of the study are the thorough statistical analysis, the exclusion of newborns with known other risk factors for hearing loss and the 1-year length of follow-up of children. Lastly, this study was mono-centric since only neonates born at the hospital were selected, greatly reducing selective reporting bias.

## 5. Conclusions

Although it seems reasonable to hypothesize that the fetal inner ear cells are damaged after the mother’s SARS-CoV-2 exposure during pregnancy, with subsequent hearing impairment, the results of our study did not strongly confirm this assumption. No cases of moderate/severe bilateral hearing loss were detected during the 1-year follow-up, while the presence of findings consistent with middle ear dysfunction was concurrently observed. It is important to clarify the possible effect of the virus on late-onset hearing loss and future research is needed.

## Figures and Tables

**Table 1:** Epidemiological features regarding mothers and newborns in total and according to the trimester of maternal infection. In the last two columns, statistical analysis is reported.

	Sample Size (n = 119)	I Trimester (n = 7) 5.8%	II Trimester (n = 45) 37.8%	III Trimester (n = 67) 56.3%	Test Significance	Post-Hoc Analyses
Female	61 (51.3%)	5 (71.4%)	23 (51.1%)	33 (49.3%)	0.543 ^a^	0.968; 0.810; 1.000 ^c^
Male	58 (48.7%)	2 (28.6%)	22 (48.9%)	34 (50.7%)		
Pregnancy						
twin	3 (2.5%)	-	-	3 (4.5%)	0.309 ^a^	1.000; 1.000; 0.426 ^c^
not physiological	13 (10.9%)	1 (14.3%)	5 (11.1%)	7 (10.4%)	0.953 ^a^	1.000; 1.000; 1.000 ^c^
complications	14 (11.8%)	1 (14.3%)	1 (2.2%)	12 (17.9%)	0.040 ^a^	1.000; 1.000; 0.035 ^c^
Age of the mother	31.55 (±5.38)	33.00 (±4.87)	31.13 (±5.88)	31.67 (±5.12)	0.670 ^b^	1.000; 1.000; 1.000 ^c^
Gestational age (weeks)	38.77 (±2.25)	40.29 (±1.11)	39.09 (±1.73)	38.40 (±2.55)	0.052 ^b^	0.559; 0.103; 0.333 ^c^
Very preterm (28 to 34 weeks)	4 (3.4%)	-	1 (2.2%)	3 (4.5%)		
Moderate to late preterm (35 to 37 weeks)	10 (8.4%)	-	2 (4.4%)	8 (11.9%)		
Weight at birth (grams)	3255.6 (±582.6)	3490.7 (±209.3)	3278.8 (±422.5)	3215.0 (±690.0)	0.469 ^b^	1.000; 0.707; 1.000 ^c^
Apgar at 1 min	8.65 (±1.65)	9.00 (±0.58)	9.04 (±1.13)	8.36 (±1.95)	0.095 ^b^	1.000; 0.971; 0.110 ^c^
Apgar at 5 min	9.66 (±0.83)	10 (±0.00)	9.83 (±0.486)	9.51 (±1.01)	0.074 ^b^	1.000; 0.395; 0.143 ^c^
Gestational age of first ABR	50.06 (±7.75)	50.00 (±4.00)	48.04 (±4.18)	51.42 (±9.47)	0.077 ^b^	1.000; 1.000; 0.072 ^c^
Maternal infection						
Symptomatic	87 (73.1%)	6 (85.7%)	33 (73.3%)	48 (71.6%)	0.731 ^a^	1.000; 1.000; 1.000 ^c^
less than 7 days	29 (33.3%)	1 (14.3%)	11 (33.3%)	17 (35.4%)	0.154 ^a^	0.167; 0.206; 1.000 ^c^
7–14 days	30 (34.5%)	1 (14.3%)	12 (36.4%)	17 (35.4%)		
more than 14 days	28 (32.2%)	4 (66.7%)	10 (30.3%)	14 (29.2%)		
Neonatal infection	2 (1.7%)	-	-	2 (3.0%)	0.461 ^a^	1.000; 0.701; 1.000 ^c^
Symptomatic	1 (0.8%)	-	-	1 (1.5%)	0.157 ^a^	-

^a^ Chi-square test; ^b^ One-way ANOVA; ^c^ Bonferroni’s post hoc analysis (I vs. II; I vs. III; II vs. III). ABR = Auditory Brainstem Evoked Response.

**Table 2:** The group of newborns with pathological ABRs.

Id	Birth				Within 3 Months				1 Month after the Audiological Assessment			
	Trimester of Maternal Infection	Gestational Age (weeks)	Weight at Birth (Grams)	OAEs Left Ear; Right Ear	ABR V Wave Threshold (dB nHL) Left; Right	OAEs Left; Right	Immittance Test ^1^ Left; Right	Otoscopy ^2^ Left; Right	ABR V Wave Threshold (dB nHL) Left; Right	Immittance Test ^1^ Left; Right	Otoscopy ^2^ Left; Right	Notes
1	I	41	3730	Pass; pass	40; 30	Pass; pass	A; A	D; N	30; 30	A; A	N; N	-
2	II	39	3530	Pass; pass	40; 40	Pass; pass	A; A	N; N	30; 30	A; A	N; N	Episode of fever and symptoms of SARS-CoV-2 infection
3	III	40	3770	Pass; pass	50; 30	Pass; pass	-	N; N	40; 30	A; A	N; N	-
4	III	38	3210	Pass; pass	40; 30	Pass; pass	A; A	D; D	40; 30	A; A	D; D	Pharyngitis; Negative oro-nasal swab
5	III	38	2880	Pass; pass	30; 50	pass; pass	A; A	N; N	30; 30	A; A	N; N	-

^1^ According to Jerger classification [23]: types A, B and C; ^2^ N: normal otoscopy; D: disorders of the middle ear such as endotympanic effusion or tympanic membrane retraction. Abbreviations: OAEs = OtoAcoustic Emissions; ABR = Auditory Brainstem Evoked response.

**Table 3:** Results of 1-year follow-up audiological assessments.

	Sample Size (*n* = 37)	I Trimester (*n* = 2)	II Trimester (*n* = 16)	III Trimester (*n* = 19)	Significance
PTA (dB)	26.39 (±3.53)	26.25 (±5.30)	27.19 (±3.15)	25.72 (±3.80)	0.227 ^b^
Immittance test ^1^					
Type A	26 (70.3%)	1 (50.0%)	11 (68.8%)	14 (73.7%)	0.773 ^a^
Type B	5 (13.5%)	0 (0.0%)	2 (12.5%)	3 (15.8%)	
Type C	6 (16.2%)	1 (50.0%)	3 (18.7%)	2 (10.5%)	
Acoustic reflexes					
present	23 (62.1%)	1 (50.0%)	12 (75.0%)	10 (52.6%)	0.138 ^a^
absent	14 (38.9%)	1 (50.0%)	4 (25.0%)	9 (47.4%)	
Otoscopy					
normal	27 (73.0%)	1 (50.0%)	12 (75.0%)	14 (73.7%)	0.300 ^a^
alterations ^2^	10 (27.0%)	1 (50.0%)	4 (25.0%)	5 (26.3%)	

^a^ Chi-square test; ^b^ One-way ANOVA test; ^1^ immittance test according to Jerger classification [23]; ^2^ disorders of the middle ear such as endotympanic effusion or tympanic membrane retraction.

## Data Availability

Raw data were generated at the Azienda Ospedaliero-Universitaria of Modena. Derived data supporting the findings of this study are available from the corresponding author on request.
